# Profiling disease experience in patients living with brain aneurysms by analyzing multimodal clinical data and quality of life measures

**DOI:** 10.1038/s41598-025-15544-1

**Published:** 2025-08-20

**Authors:** Sebastian R. Reder, Jochen Hardt, Marc A. Brockmann, Carolin Brockmann, Suam Kim, Melina Kawulycz, Marie Schulz, Sven Rainer Kantelhardt, Katja Petrowski, Sabine Fischbeck

**Affiliations:** 1 Department of Neuroradiology, University Medical Center, Johannes Gutenberg-University of Mainz, Mainz, Germany; 2 Department of Medical Psychology and Medical Sociology, University Medical Center of the Johannes Gutenberg-University Mainz, Mainz, Germany; 3 Department of Neurosurgery, Vivantes Hospital Friedrichshain, Berlin, Germany

**Keywords:** Aneurysm, Intracranial, Quality of life, Population, Vulnerable, Models, Biopsychosocial, Vasospasm, Brain, Brain injury, Chronic, Mental health, Return to work, Care, Patient-Centered, Care Planning, Patient, Brain injuries, Cerebrovascular disorders, Neurovascular disorders, Stroke

## Abstract

To explore the mental and physical health (MH, PH) on individuals living with brain aneurysms and to profile their differences in disease experience. In *N* = 111 patients the Short Form 36 Health Survey (SF-36) was assessed via an online survey; Supplementary data included angiography and magnetic resonance imaging (MRI) findings (including AI-based brain Lesion Volume analyses in ml, or LV). Correlation and regression analyses were conducted (including biological sex, age, overall brain LV, PH, MH). Disease profiles were determined using principal component analysis. Compared to the German normative cohort, patients exhibited overall lower SF-36 scores. In regression analyses, the DW was predictable by PH (β = 0.345) and MH (β=-0.646; *R* = 0.557; *p* < 0.001). Vasospasm severity correlated significantly with LV (*r* = 0.242, *p* = 0.043), MH (*r*=-0.321, *p* = 0.043), and PH (*r*=-0.372, *p* = 0.028). Higher LV were associated with poorer PH (*r*=-0.502, *p* = 0.001), unlike MH (*p* > 0.05). Main disease profiles were identified: (1) those with increased LV post-rupture (high DW); (2) older individuals with stable aneurysms (low DW); (3) revealing a sex disparity in QoL despite similar vasospasm severity; and 4), focused on chronic pain and its impact on daily tasks. Two sub-profiles highlighted trauma-induced impairments, functional disabilities from LV, and persistent anxiety. Reduced thalamic and pallidal volumes were linked to low QoL following subarachnoid hemorrhage. MH has a greater impact on life quality compared to physical disabilities, leading to prolonged DW. A singular physical impairment was rather atypical for a perceived worse outcome. Patient profiles revealed that clinical history, sex, psychological stress, and pain each contribute uniquely to QoL and work capacity. Prioritizing MH in assessing workability and rehabilitation is crucial for survivors’ long-term outcome.

**Supplementary Information:**

The online version contains supplementary material available at 10.1038/s41598-025-15544-1.

## Background

Aneurysmal subarachnoid hemorrhage (aSAH) is a distinct form of hemorrhagic stroke that constitutes approximately 5% of all stroke cases in most Western nations^1^. It predominantly affects individuals with a mean age ranging from 50 to 60^2,3^. Despite a relatively high initial survival rate following the hemorrhage, up to 30% of the patients experience neurological deterioration due to delayed cerebral ischemia (DCI) associated with SAH^4^. The precise pathophysiology of DCI remains understood incompletely but is believed to involve a multifaceted interplay of factors including large vessel vasospasm (LVV), microcirculatory dysfunction, cortical spreading depolarization, and inflammation^5,6^. Treating isolated radiographically identifiable cerebral LVV does not cause improved outcomes consistently, thereby highlighting the complexity of pathophysiological interactions^7,8^. All these factors contribute to increased damage to the brain parenchyma, resulting in diminished physical functionality and the persistence of lesions in cerebral MR imaging (cMRI) detectable years after the primary event^9,10^.

A connection between psychiatric disorders and stroke, particularly in patients with aneurysms who are not routinely screened for such risk factors, is increasingly recognized^11^. Additionally, neuroendocrine disruptions contribute significantly to an impaired quality of life and sleep disturbances in subarachnoid hemorrhage (SAH) patients^12^, leading to an elevated prevalence of depression, anxiety and post-traumatic stress disorder (PTSD), persisting even months after the traumatic event^13^. Notably, those who have experienced an SAH and still harbor an unsecured cerebral aneurysm tend to experience even higher levels of anxiety^13^. Furthermore, a decrease in the functional status among aneurysm patients is strongly associated with depression and diminished overall mental well-being^13^. All these factors contribute to an increased risk and make individuals more likely to encounter difficulties in returning to work^14^.

In the context of this study, the Short Form-36 Health Survey (SF-36), an established questionnaire assessing various dimensions of physical and mental health was administered and assessed online one year after discharge. The associated information was correlated with cerebral MRI using AI-based brain parenchymal and lesion volumetry as well as neurointerventional Digital Subtraction Angiography (DSA) findings from primary aneurysm management. The aim of the study was to correlate SF-36 responses objectively with clinical data and brain parenchymal changes, aiming to identify multimodal risk factor profiles using physical and mental health aspects associated with increased work disability. It was hypothesized that persistent fear of rupture could cause increased mentally-induced physical impairments. On the other hand, the trauma associated with treating an acutely ruptured aneurysm could result in heightened physical and mental damage, both potentially contributing to increased disability.

## Materials and methods

### Ethical and large language model statement

The authors guarantee the study was conducted in compliance to the Declaration of Helsinki from 1964 and to the local ethical guidelines. All experimental protocols are approved by the Ethics Committee of the Landesärztekammer Rheinland-Pfalz [engl.: “State Medical Association of Rhineland-Palatinate”] regarding the inclusion of aneurysm patients (number: 837.366.17, 1120) or the application of Patient Related Outcome Measures (PROMs) in neurosurgical patients (number: 837.097.15, 9865). After a study explanation, informed consent was obtained from participants or their legal guardians.

Large Language Models (LLMs) were used for the grammatical correction of the manuscript. These corrections were verified by the authors.

### AneuryCare study recruitment

**Fig. 1:**
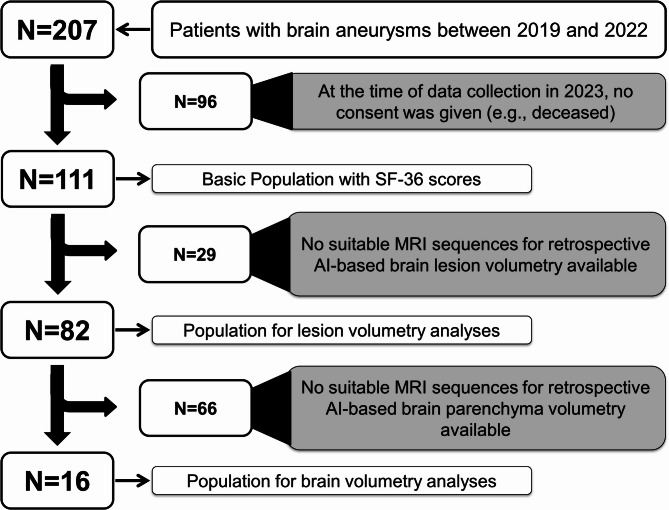
Patient population selection.

All patients with brain aneurysms who were discussed by a neurovascular expert board during routine clinical care between 2019 and 2022 were identified (*n* = 207; see Fig. 1). After a telephone explanation of the planned “AneuryCare” study, informed consent could not be obtained from 96 individuals, (primarily due to death). For 111 patients, basic demographic data and neurointerventional DSA findings were available for analysis. In 82 of these patients, suitable MRI scans could be obtained—sourced both from external radiology practices and the hospital—for retrospective volumetry of brain lesions. Finally, due to limited availability of appropriate MRI sequences, AI-based volumetry of subcortical brain parenchymal structures was possible in only 16 patients.

### Basic population parameters

The online survey presented to participants was structured into multiple sections to systematically collect data on demographics, health-related quality of life, and the psychosocial consequences of being diagnosed with a brain aneurysm. Initially, respondents were prompted to enter fundamental demographic information such as their age and sex. In the online questionnaire, work disability in weeks per year (DW) was assessed with the following question: “How long after your hospital stay did you not work or were you unable to work? (in weeks per year)”.

### Short Form-36 health survey

The online SF-36 questionnaire assessed eight domains—four pertaining to physical health and four to mental health—alongside demographic variables such as sex and age^15^. Each SF-36 domain yields a quality of life (QoL) score, standardized on a scale from 0 (indicating no QoL), through 50 (representing the population average), to 100 (reflecting exceptionally high QoL). SF-36 scores were transformed into percentile values to facilitate direct comparison across different population groups, i.e. the German normal population. This standardization establishes a consistent metric for evaluating health status, thereby supporting meaningful benchmarking with reference populations.

### Physical domain

**Physical Functioning (PhyFunc)** evaluates the impact of the health condition on activities like self-care, walking, climbing stairs, bending, lifting, and moderate or vigorous activities.

**Physical Role Function (PhyRoFunc)** assesses how the health condition affects work or daily activities, including reduced productivity, activity limitations, or difficulties in certain tasks.

**General Health (GenH)** evaluates personal perceptions of health, encompassing current health status, future outlook, and ability to cope with illness and its aftermath.

**Pain-related Quality of Life (Pain)** measures the intensity of pain and its interference with normal activities, both at home and elsewhere.

### Mental domain

**Social Functioning (SoFunc)** evaluates the extent to which physical health or emotional issues impair normal social activities (i.e. family life).

**Emotional Role Function (EmRoFunc)** assesses the extent to which emotional problems affect work or other daily activities, including spending less time, accomplishing less, and not working as effortlessly as usual.

**Vitality (Vit)** assesses feelings of being energized and full of vigor versus tired and exhausted.

**Psychological Well-Being (PsyWB)** encompasses overall mental health, including depression, anxiety, emotional and behavioral control, and general positive mood.

### Findings from the neurointerventional digital subtraction angiography

The DSA-related written report included information on the status of the brain aneurysm (ruptured vs. unruptured), the occurrence and severity of LVV (rated from 0 = no spasms to 4 = severe), and whether residual perfusion within the aneurysm could be delineated after treatment (see Fig. 1; Table 1).

**Table 1:** Basic population characteristics.

		Women	*N* = 83		Men	*N* = 28	
Variable	Subgroup	Mean/N	±SD	Missing	Mean/N	±SD	Missing
Age		61.976	12.295	0	61.250	11.527	0
Ruptured Aneurysm	No	70		0	24		0
	Yes	13			4		
Vasospasm Severity	No (1)	70		0	24		0
	Minor (2)	5		0	0		0
	Moderate (3)	4		0	1		0
	Severe (4)	4		0	3		0
Residual Aneurysm Perfusion	No	40			17		
	Yes	43		0	11		0
Working Inability [Weeks/Year]		8.545	17.120	39	6.000	14.376	15
Brain Lesion Volume [ml]	Global	9.803	11.564	19	9.416	10.766	10
Brain Parenchyma Volume [ml]	Left Caudate Nucleus	2.597	0.957	68	1.783	1.004	25
	Right Caudate Nucleus	2.890	1.060	68	1.933	0.544	25
	Left Putamen	3.393	1.130	68	2.940	0.702	25
	Right Putamen	3.398	1.086	68	2.630	0.602	25
	Left Pallidum	1.147	0.375	68	1.127	0.075	25
	Right Pallidum	1.175	0.374	68	1.090	0.089	25
	Left Thalamus	6.713	1.890	68	6.487	0.732	25
	Right Thalamus	6.751	1.679	68	6.367	0.869	25

### Cerebral MR imaging with brain lesion and brain parenchyma volumetry

The MRI data were obtained as part of the clinical routine during the 1-year follow-up examination after the initial discussion in the neurovascular expert board. Image data were acquired using scanners with field strengths of 1.5 or 3 Tesla within the primary care facility, or referring hospitals. The commercial AI-based software “mdbrain” (Version 4.13.0; Mediaire GmbH, Berlin, Germany; https://mediaire.ai/en/mdbrain/) was used for the evaluation of MR images. Two MR sequences were analyzable: (1) brain lesion volumetry and (2) brain volumetry. For lesion volumetry, T2-weighted datasets (T2w TSE, T2w FLAIR, or T2w SPACE) were required, with orientation being inconsequential (axial, sagittal, or coronal). Due to heterogeneous MRI protocols, *n* = 82 datasets were available in-house and externally to assess the quantity of the so called “white matter lesions”. Brain parenchymal volumetry necessitated T1-weighted 3D datasets (T1w MPRAGE), for which only *n* = 16 MRIs were appropriate. This function allowed for identifying deep brain structures and corresponding brain volume, such as the thalami.

### Statistical analyses

All analyses were conducted using the statistical software SPSS (Version 29; IBM, Armonk, NY, USA; https://www.ibm.com/products/spss-statistics).

To identify potential risk factor profiles for long-term working disability, principal component analyses (PCA) were performed using a rotated component matrix (Varimax-Rotation Method with Kaiser-Meyer-Olkin normalization and Barlett’s Test of sphericity). Varimax rotation was applied to simplify the factor structure by maximizing the variance of squared loadings and reducing cross-loadings. The Kaiser-Meyer-Olkin (KMO) measure was used to assess sampling adequacy, while Bartlett’s Test of Sphericity was used to confirm that the correlation matrix was appropriate for factor extraction. Together, these methods were used to minimize errors such as factor indeterminacy, multicollinearity, and the extraction of unreliable factors, thereby producing a more robust and interpretable factor solution.

Correlations between SF-36 dimensions and other ordinal-scaled parameters were examined using Spearman’s Rank Correlation Coefficient Test. To account for the increased risk of Type I error due to multiple testing, the Bonferroni correction was applied to adjust the significance threshold accordingly. For measuring nominal scaled variables, chi-square tests were employed, utilizing the Fisher correction. The use of chi-square tests with Fisher correction was intended to reduce errors associated with small sample sizes and low expected cell frequencies. Specifically, these methods were used to minimize the risk of Type I errors (false positives) and to ensure the validity of statistical conclusions when analyzing nominally scaled variables. Mediation analysis revealed no significant interaction among the subgroups^16^, and no relevant interactions were observed among the subgroups. Multicollinearity was assessed with a correlation threshold of *r* > 0.9 (*p* < 0.05)^17^.

For the data analysis, multivariate linear regression with backward elimination and stepwise variable selection was used to predict the disability in weeks per year (DW) including age, sex, MH, and PH. In this process, the regression coefficients (R, B, 95% CI for B, and standardized β; including p-values), the determination coefficient R² (or “R squared”) were provided. Only regression models with predictors with statistically significant influences on the regression equations were reported (*p* < 0.05). For a statistical power of 1-β = 0.9 with a R²=0.6, an alpha level of α = 0.05 and three predictors for multivariate regression analysis the collective should contain *n* = 15 subjects at minimum^18^. Meanwhile using two predictors in the same setting, *n* = 13 subjects would be sufficient to achieve a statistical power of 1-β = 0.9^18^.

## Results

### Cohort characteristics

This study included n=83 women and n=28 men (61.97±12.29 years vs. 61.25±11.527 years; see Table 1). Ruptured aneurysms were observed in n=13 women (16%) and n=4 men (14%), while severe vasospasms following rupture were evident in n=4 women and n=3 men (5% vs. 10%). The cerebral lesion volume in women and men was approximately equal (9.8 ± 11.5 vs. 9.4 ± 10.8). Women experienced longer periods of work disability than men (8.5 ± 17.1 weeks vs. 6.0 ± 14.4 weeks).

### Standardized Short Form 36-Scores

The AneuryCare population showed following scores in the SF-36: PhyFunc=68.6; PhysRoFunc=37.79; GenH=69.53; Pain=51.07; SoFunc=45.16; EmRoFunc=69.88; Vit=52.05; PsyWB=61.08.

### Regression analysis

In regression analyses, the DW was predictable by MH (β = – 0.646; 95% CI – 0.991 to – 0.299) and PH (β=0.345 with 95% CI – 0.0018 to 0.6917 R=0.557; *p*<0.001; see Supplemental Figure 1).

### Correlation analyses

The correlation analyses revealed varying strengths of association between clinical variables and SF-36 quality of life domains (Table 4). The correlation between longer DW and reduced pain-related quality of life (r = – 0.273; *p* = 0.04) represents a minor association, while the relationship with reduced emotional role function (EmRoFunc; r = – 0.406; *p* = 0.002) is moderate. Differences in physical and mental quality of life between men and women (r = 0.246 to 0.298; *p* = 0.05 to 0.01) also indicate minor to moderate correlations. The negative association between higher age and physical quality of life (r = – 0.417; *p* = 0.001) is moderate. The impact of the occurrence of an aneurysm rupture and subsequent vasospasm on quality of life, both in physical (r = –0.266 to –0.310; *p* = 0.03 to 0.01) and mental domains (r =–0.242 to – 0.312; *p*=0.05 to 0.01), ranges from minor to moderate. Higher brain lesion volumes were associated with minor to strong negative correlations in physical quality of life (r = – 0.284 to –0.509; *p* = 0.05 to <0.001) and a moderate negative correlation with psychological well-being (r = – 0.314; *p*=0.03). Notably, decreased vitality in the mental quality of life domain was strongly associated with increased volumes in the left pallidum (r = – 0.741; *p* = 0.01) and left thalamus (r = – 0.684; *p* = 0.02). Additionally, psychological well-being and pain-related quality of life showed strong positive correlations with the volume of the right thalamus (r = 0.591 and 0.625; *p*=0.05 each). Residual aneurysm perfusion showed no significant correlation with any SF-36 dimension. Overall, the observed correlations ranged from minor to strong, depending on the specific domain and clinical factor analyzed.

### Patient profile identification

#### Principal components analysis using demographic and clinical data, as well as PH/MH-Sumscores

For evaluating potential risk profiles leading to increased work disability, a PCA was conducted. Principal component analyses incorporated a set of clinical and demographic variables, including duration of work disability, sex, age, occurrence of aneurysm rupture, vasospasm severity, brain lesion volume, and residual blood flow within the aneurysm. Additionally, the sum scores of the SF-36 physical and mental health domains (PH, MH) were included. This multivariate approach enabled the identification of initial patient profiles (Table 2), illustrating the interplay between medical, demographic, and patient-reported health factors in relation to work incapacity and quality of life. The Kaiser–Meyer–Olkin measure of sampling adequacy yielded a value of 0.681, suggesting a relatively robust factor analysis, while Bartlett’s Test of sphericity was significant (*p*<0.001), affirming that correlations among variables were suitable for PCA. According to Guttman’s and Kaiser’s criteria (1954 and 1969), only factors with eigenvalues ≥ 1 were included. Analyses based on Kaiser’s criteria and the scree plot indicated the retention of three factors (here: Profile 1-3) with eigenvalues exceeding 1, accounting for 70.2% of the total variance.

**Table 2:** Profiling brain aneurysm patients by using principal components analysis.

Variables [Units]	Subgroup	Profile 1	Profile 2	Profile 3
Working Inability [Weeks/Year]		0.297	-0.696	-0.120
Sex	Male = 1	0.074	0.164	0.908
Age		0.100	0.778	0.073
Ruptured Aneurysm	Yes = 1	0.876	-0.091	0.177
Vasospasm Severity	Severe = 4	0.918	-0.135	0.229
Brain Lesion Volume [ml]	Global	0.727	-0.201	-0.097
Residual Aneurysm Perfusion	Yes = 1	-0.543	0.422	-0.159
SF-36 Physical Domain (Well-being)	Sumscore	-0.800	-0.197	0.396
SF-36 Mental Domain (Well-being)	Sumscore	-0.687	0.013	0.452
*Measures of Sampling Adequacy*				
*Kaiser-Meyer-Olkin Value*	*0.681*			
*Barlett Test (p-Value)*	*< 0.001*			
*Explained Variance [Cumulative]*	*70.2%*			

This analysis revealed that moderately elevated work disability was strongly correlated with aneurysm rupture, increased lesion volume as well as reduced physical and mental quality of life (see Table 2, Profile 1). In this profile, residual aneurysm perfusion had a moderate to strong inverse effect.

Profile 2 exhibited a strong correlation between reduced work disability at older ages and residual aneurysm perfusion (Table 2). No significant influence on physical or mental health was observed.

In Profile 3 (Table 2), the female sex (coded as 0) was strongly associated with reduced physical and mental health, while also exhibiting moderate vasospasms. Conversely, in men (coded as 1) with moderate vasospasms, a moderately preserved physical and mental QoL was observed.

#### Principal components analysis using eight SF-36 dimensions

For further sub-classification and identifying sub-profiles (S1-4), a PCA was conducted, considering each of the eight dimensions of physical and mental QoL from the SF-36 (Table 3). The Kaiser–Meyer–Olkin measure of sampling adequacy yielded a value of 0.734 and Bartlett’s Test of sphericity was significant (p<0.001), affirming that correlations among variables were suitable for PCA. Only factors with eigenvalues ≥ 1 were included, according to Guttman’s and Kaiser’s criteria (1954 and 1969). Analyses based on Kaiser’s criteria and the scree plot indicated the retention of four factors (here: Profile S1-4) with eigenvalues exceeding 1, accounting for 75.4% of the total variance.

In Profile S1 (Table 3), work disability exhibited a strong loading inverse to residual aneurysm perfusion, and the QoL regarding physical role function (PhyRoFunc), vitality (Vit), psychological well-being (PsyWB), emotional role function (EmRoFunc), and social functioning (SoFunc).

**Table 3:** Profiling with SF-36 and other potential risk factors for working inablility.

Variables [Units]/(Subgroups)	Profile S1	Profile S2	Profile S3	Profile S4
Working Disability [Weeks/Year]	– 0.877	0.059	0.139	0.012
Vasospasm Severity (Severe = 4)	– 0.246	0.861	– 0.136	0.031
Brain Lesion Volume [ml]	– 0.082	0.814	– 0.199	0.054
Residual Aneurysm Perfusion (Yes = 1)	0.420	– 0.617	– 0.350	0.207
Physical Domain: Physical Role Function	0.420	– 0.254	0.538	0.427
Physical Domain: Pain-related Quality of Life	0.035	0.009	– 0.072	0.919
Physical Domain: General Health	– 0.037	– 0.113	0.745	0.123
Physical Domain: Physical Functioning	0.099	– 0.703	0.415	0.295
Mental Domain: Vitality	0.458	– 0.084	0.466	0.579
Mental Domain: Psychological Well-being	0.434	– 0.251	0.706	0.061
Mental Domain: Emotional Role Function	0.781	– 0.124	0.410	– 0.030
Mental Domain: Social Functioning	0.681	– 0.343	0.318	0.294
*Measures of Sampling Adequacy*				
*Kaiser-Meyer-Olkin Value*	0.734			
*Barlett Test (p-Value)*	< 0.001			
*Explained Variance [Cumulative]*	75.4%			

In Profile S2 (Table 3), a strong loading of vasospasm severity was observed with the degree of brain lesions, concurrently exhibiting an inverse loading on physical functionality (PhyFunc) and aneurysm perfusion. Moderate loading into the profile was contributed by the dimensions of psychological well-being (PsyWB) and social functioning (SoFunc) of the mental domain, inverse to vasospasm severity and brain lesion load.

In Profile S3 (Table 3), moderate to strong effects were evident across seven of the eight dimensions (excluding Pain-related QoL), concurrently with inversed effects on aneurysm perfusion (Table 4).

**Table 4:** Correlation of physical and mental dimensions with DSA- and MRI-based parameters.

		Physical Domain: Physical Role Function	Physical Domain: General Health	Physical Domain: Physical Functioning	Physical Domain: Pain-related Quality of Life	Mental Domain: Vitality	Mental Domain: Psychological Well-being	Mental Domain: Emotional Role Function	Mental Domain: Social Functioning
Working Inability [Weeks/Year]	R	0.043	– 0.026	0.043	**– 0.273**	– 0.171	– 0.111	**– 0.406**	**– 0.300**
	P	0.74	0.84	0.74	**0.04**	0.20	0.41	**0.002**	**0.02**
Sex (male = 1)	R	**0.285**	0.107	**0.285**	**0.298**	**0.246**	0.103	0.220	0.180
	P	**0.02**	0.40	**0.02**	**0.01**	**0.05**	0.42	0.08	0.16
Age	R	**– 0.417**	0.133	**– 0.417**	– 0.169	0.018	– 0.005	– 0.003	– 0.072
	P	**0.001**	0.30	**0.001**	0.18	0.88	0.96	0.97	0.57
Ruptured Aneurysm (Yes = 1)	R	**– 0.310**	**– 0.266**	**– 0.310**	– 0.062	**– 0.242**	– 0.238	**– 0.255**	**– 0.312**
	P	**0.01**	**0.03**	**0.01**	0.63	**0.05**	0.06	**0.04**	**0.01**
Vasospasm Severity (Severe = 4)	R	**– 0.317**	**– 0.267**	**– 0.317**	– 0.062	– 0.239	– 0.239	**– 0.256**	**– 0.314**
	P	**0.01**	**0.03**	**0.01**	0.63	0.06	0.06	**0.04**	**0.01**
Brain Lesion Volume [ml]	R	**– 0.509**	– 0.096	**– 0.509**	**– 0.284**	– 0.076	**– 0.314**	– 0.240	– 0.260
	P	**< 0.001**	0.52	**< 0.001**	**0.05**	0.61	**0.03**	0.10	0.07
Residual Aneurysm Perfusion	R	0.121	– 0.071	0.121	0.055	0.176	0.137	0.163	0.239
	P	0.34	0.58	0.34	0.67	0.17	0.29	0.20	0.06

The analysis incorporated baseline population data such as working inability in weeks/year, age, sex (0 = female; 1 = male), along with angiographic (DSA) and CT-derived parameters: aneurysm rupture status (0 = no; 1 = yes), vasospasm severity (0 = none to 4 = severe), and residual aneurysm perfusion detection (0 = no; 1 = yes). Additionally, MRI-based brain lesion volumes from *n* = 82 subjects were included. These variables were correlated with SF-36 subdomain scores using spearman’s rank correlation. Results were reported as the spearman coefficient (R) and significance level (p), with adjustments for multiple comparisons using the bonferroni correction to mitigate type I error risks.

In Profile S4 (Table 3), pain-related QoL predominantly loaded with consistently moderate to high effects on physical role function (PhyRoFunc), physical functioning (PhyFunc), vitality (Vit), and social functioning (SoFunc).

## Discussion

**Fig. 2:**
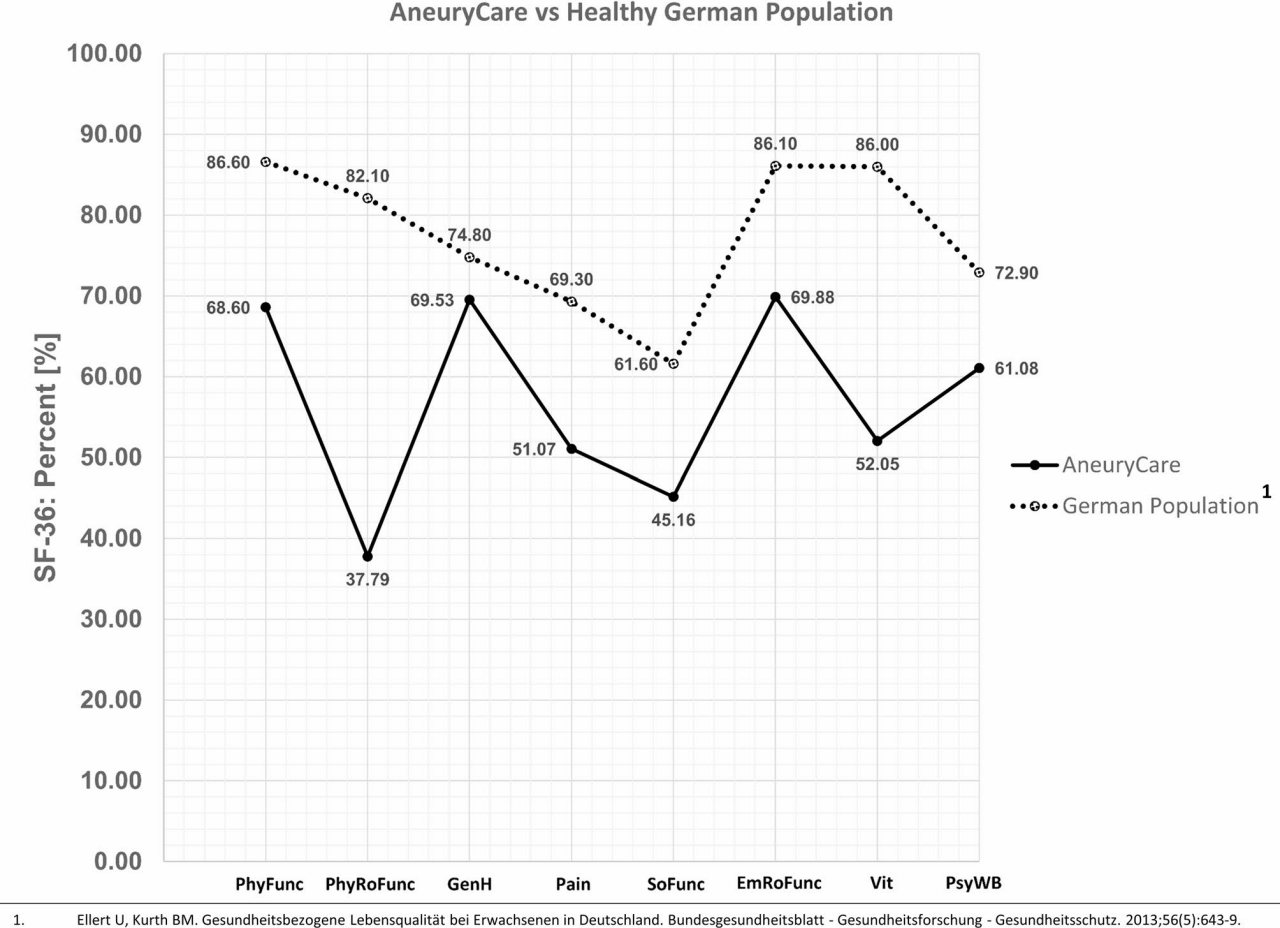
Comparison of the SF-36 scores of the AneuryCare group to a healthy German normative cohort. Physical Functioning (PhyFunc) measures the impact of the health condition on basic activities like self-care, walking, and exercise. Physical Role Function (PhyRoFunc) assesses how the condition affects work and daily tasks, including limitations and reduced productivity. General Health (GenH) reflects personal perceptions of overall health and ability to cope with illness. Pain-related Quality of Life (Pain) evaluates pain intensity and its interference with daily activities. Social Functioning (SoFunc) measures how physical or emotional health issues affect social activities, such as family life. Emotional Role Function (EmRoFunc) evaluates how emotional problems impact work or daily activities, including reduced time spent and decreased productivity. Vitality (Vit) assesses feelings of energy and vigor versus fatigue and exhaustion. Psychological Well-Being (PsyWB) reflects overall mental health, including depression, anxiety, emotional control, and general mood.

**Fig. 3:**
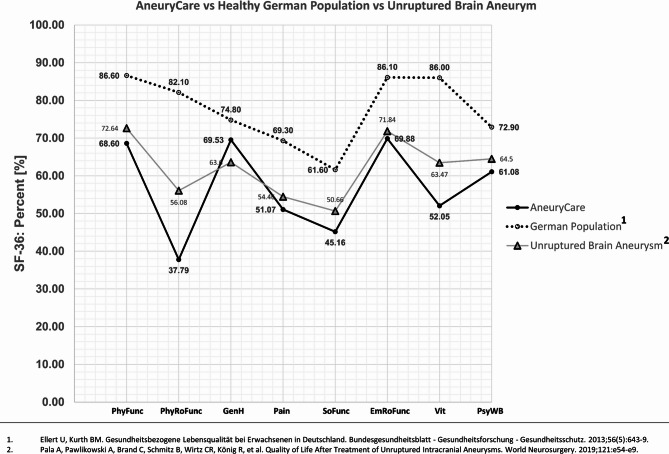
Comparison of the SF-36 scores of the AneuryCare group to a healthy German normative cohort, and a study analyzing patients with unruptured brain aneurysms. Physical Functioning (PhyFunc) measures the impact of the health condition on basic activities like self-care, walking, and exercise. Physical Role Function (PhyRoFunc) assesses how the condition affects work and daily tasks, including limitations and reduced productivity. General Health (GenH) reflects personal perceptions of overall health and ability to cope with illness. Pain-related Quality of Life (Pain) evaluates pain intensity and its interference with daily activities. Social Functioning (SoFunc) measures how physical or emotional health issues affect social activities, such as family life. Emotional Role Function (EmRoFunc) evaluates how emotional problems impact work or daily activities, including reduced time spent and decreased productivity. Vitality (Vit) assesses feelings of energy and vigor versus fatigue and exhaustion. Psychological Well-Being (PsyWB) reflects overall mental health, including depression, anxiety, emotional control, and general mood.

**Fig. 4:**
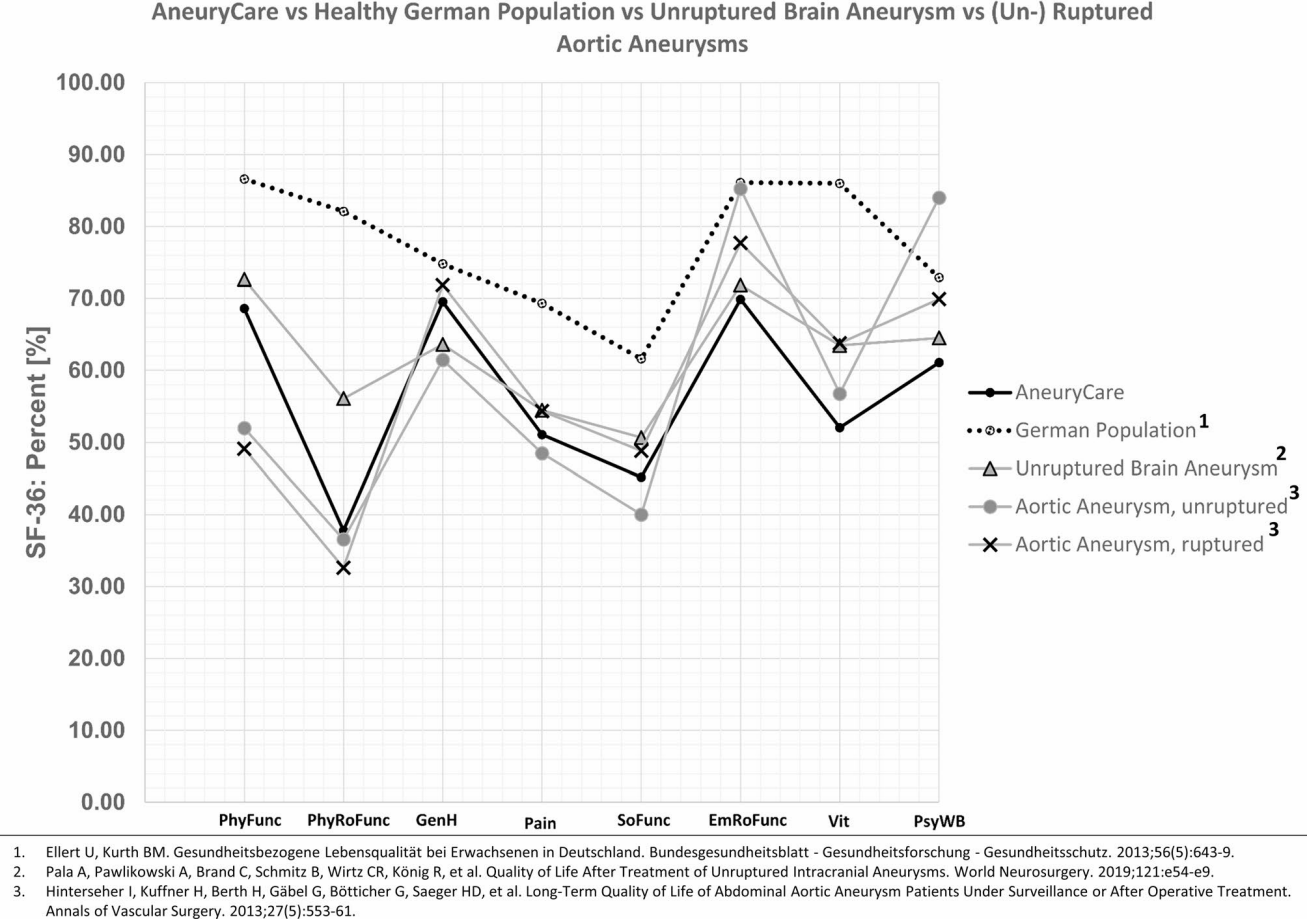
Comparison of the SF-36 scores of the AneuryCare group to a healthy German normative cohort, a study analyzing patients with unruptured brain aneurysms, and patients with (un-) ruptured aortic aneurysms. Physical Functioning (PhyFunc) measures the impact of the health condition on basic activities like self-care, walking, and exercise. Physical Role Function (PhyRoFunc) assesses how the condition affects work and daily tasks, including limitations and reduced productivity. General Health (GenH) reflects personal perceptions of overall health and ability to cope with illness. Pain-related Quality of Life (Pain) evaluates pain intensity and its interference with daily activities. Social Functioning (SoFunc) measures how physical or emotional health issues affect social activities, such as family life. Emotional Role Function (EmRoFunc) evaluates how emotional problems impact work or daily activities, including reduced time spent and decreased productivity. Vitality (Vit) assesses feelings of energy and vigor versus fatigue and exhaustion. Psychological Well-Being (PsyWB) reflects overall mental health, including depression, anxiety, emotional control, and general mood.

In comparison to a healthy German normative cohort, the SF-36 scores were lower in the AneuryCare group (Fig. 2), which included patients with ruptured, unruptured, and electively treated brain aneurysms^19^. A particularly notable decline was observed in the domains of physical role functioning (PhyRoFunc) and vitality (Vit). In a separate study of patients with unruptured brain aneurysms (Fig. 3), every SF-36 dimension was lower in the AneuryCare group, with the exception of general health (GenH)^20^. When compared to patients with unruptured and ruptured aortic aneurysms^21^, as well as unruptured brain aneurysms^20^, a roughly similar profile across the SF-36 dimensions was observed (Fig. 4).The current study utilized an online version of the SF-36 Health Survey to evaluate physical and mental health dimensions in cerebral aneurysm patients, correlating them with AI-based cerebral MRI volumetry and neurointerventional DSA findings to identify multimodal risk factors for increased work disability. The SF-36 assesses how patients subjectively evaluate their own health status and situation: Mental health (MH) demonstrated a clear and significant impact: the higher MH, the lower DW. In contrast, physical health (PH) alone was not a meaningful predictor. This was further reflected in the reported model, where the confidence interval for the standardized beta of PH included zero at its lower bound, indicating a lack of statistical significance, even though the t-value was different from zero. This may suggest a relative influence of physical health on DW, while MH—potentially through a different subjective appraisal of the situation—had a stronger impact. When combining both SF-36 domains in the regression model, a moderate effect size was observed, which may indicate the presence of additional confounders. The evaluation of SF-36 data alone seems not sufficient to infer disease perception or the risk of impending work disability.

Profile 1 (Fig. 3) could categorize patients with increased residual brain parenchymal damage following an acute aneurysm rupture^22^ or elective occlusion^23,24^. Extended durations of work incapacity, possibly originating from acute care and rehabilitation interventions, were linked to reduced levels of physical and mental QoL^25,26^. As part of acute care measures, these patients underwent interventional occlusion of their acutely ruptured aneurysm^22,27–29^.

**Fig. 5:**
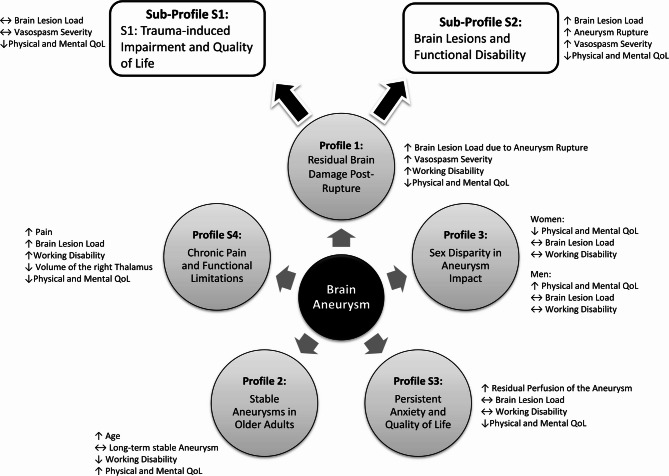
Profiling Patients living with Brain Aneurysms. “*Residual Brain Damage Post-Rupture*” characterizes patients with significant residual brain parenchymal damage following an acute aneurysm rupture, associated with prolonged work incapacity and reduced physical and mental Quality of Life (QoL). This group includes patients who underwent interventional occlusion of their acutely ruptured aneurysms. The first associated Sub-Profile “*Trauma-induced Impairment and Quality of Life*” includes individuals whose traumatic experiences cause emotional and psychomotor impairment, affecting their daily tasks. The second associated Sub-Profile “*Brain Lesions and Functional Disability*” focuses on patients with increased brain lesion burden correlating with the severity of vasospasm. These patients experience reduced physical functionality, particularly in performing moderate to heavy tasks and self-care abilities, impacting their overall QoL. “*Sex Disparity in Aneurysm Impact*” highlights a sex disparity where women report lower physical and mental QoL despite similar vasospasm severity compared to men, suggesting that women tend to exhibit heightened emotional responses to stress, influencing their self-assessed QoL. “*Persistent Anxiety and Quality of Life*” encompasses individuals with monitored residual perfused aneurysms experiencing diminished physical and mental QoL. Anxiety over living with an untreated aneurysm leads to self-imposed activity reduction, impacting their QoL but not strongly associated with prolonged work disability. “*Stable Aneurysms in Older Adults*” encompasses older individuals with long-term stable aneurysms who do not require treatment, experiencing reduced work disability due to watchful waiting strategies. “*Chronic Pain and Functional Limitations*” addresses the impact of chronic pain on QoL, correlating with decreased capacity for daily tasks and heightened fatigue. These patients exhibit psychomotor inhibition, affecting their functionality in familial and social contexts.

- Overview of Profile 1: “Residual Brain Damage Post-Rupture”.
  - Patients with increased residual brain parenchymal damage post-aneurysm rupture.
  - Prolonged work incapacity linked to reduced physical and mental QoL.
  - Interventional occlusion of acutely ruptured aneurysms as part of acute care.

Profile 2 (Fig. 5) may encompass older individuals with long-term stable aneurysms, without the necessity for treatment^24,30^. This might cause reduced work disability due to watchful waiting strategies, given the absence of widely accepted guidelines for unruptured aneurysms according to patients over 80 years of age^31,32^.

- Overview of Profile 2: “Stable Aneurysms in Older Adults”.
  - Older individuals with long-term stable aneurysms.
  - No need for treatment, leading to reduced work disability due to watchful waiting.

In Profile 3 (Fig. 5), a sex disparity emerges: despite similar severity of vasospasms, women reported lower physical and mental quality of life than men, as confirmed by correlation analyses. This might be attributable to self-assessed sex-specific evaluations of their own situation regarding different stressors^33,34^. It is evident, that women tended to exhibit heightened emotional responses to stressful situations, with men showing a greater physiological response^34,35^.

- Overview of Profile 3: “Sex Disparity in Aneurysm Impact”.
  - Women reported lower physical and mental QoL despite similar vasospasm severity.
  - Correlated with sex-specific evaluations of their situation.

From Profile 1 and correlation analyses, it is evident that a reduced aneurysm perfusion can be addressed in the context of traumatic experiences^29^. From this, Sub-Profile S1 could include individuals experiencing functional impairment in their abilities, overwhelming them emotionally and psycho-motorically (Vitality) in everyday tasks^36^. The extent of reduced physical QoL is limited to only one dimension. However, these few limitations seem to already cause a mutual and coherent relationship with three dimensions of mental QoL^36^. Mental well-being is not primarily affected, as it would reflect deficits similar to those from the spectrums of depression and anxiety. This profile’s statement can be assigned to that of Profile 1 (Fig. 3).

- Overview of Profile S1: “Trauma-induced Impairment and Quality of Life”.
  - Impairment from traumatic experiences affecting emotional and psychomotor abilities.
  - Reduced physical QoL with coherent relationship to mental QoL dimensions.

An increased brain lesion burden correlates with the severity of vasospasm, reflecting the trauma experienced by patients in Sub-Profile S2^22^. In this group, physical functionality is reduced, pertaining to the performance of solving moderate to heavy tasks and to self-care abilities (e.g., bending and lifting heavy shopping bags)^22,37^. Here, there is a reduced QoL in dimensions reflecting one’s functional ability within the family as well as those indicating depression or anxiety disorders (psychological well-being)^37,38^. This profile’s statement can be assigned to that of Profile 1 Fig. 3).

- Overview of Profile S2: “Brain Lesions and Functional Disability”.
  - Increased brain lesion burden correlates with vasospasm severity.
  - Reduced physical functionality, affecting tasks and self-care abilities.

Sub-Profile S3 (Fig. 5) could encompass individuals with primarily monitored residual perfused aneurysms, concurrently experiencing diminished physical and mental QoL^39,40^. This might be attributable to the persistent apprehension of living “with a bomb in my head”. These individuals reduce their activities to mitigate the risk of rupture, often resulting in significant physical and cognitive limitations^39,41^. Conversely, these patients might experience relief upon closure of their aneurysm^41^. Without a relevant influence of brain lesions in this profile, strongly correlating with a rupture, it is probable that these individuals suffer from primarily non-critical and acutely treatable aneurysms predominantly managed under observation^39,40^. Consequently, this would cause a self-imposed reduction in overall QoL as a form of self-protection and subsequently chronic stress^38^:

- Overview of Profile S3: “Persistent Anxiety and Quality of Life”.
  - Individuals with primarily monitored residual perfused aneurysms.
  - Experience diminished physical and mental QoL, possibly due to self-protection.

Sub-Profile S4 (Fig. 5) subsumes the impact of pain on the QoL. Here, it is evident that increased pain correlates with decreased capacity to carry out daily tasks, encompassing moderate to strenuous activities^42^. Furthermore, individuals report heightened fatigue and, as a consequence of chronic pain, exhibit psychomotor inhibition, resulting in impaired functionality within familial or social contexts^43,44^. A potential correlate of perceived pain was observed in correlation analyses: reduced pain-related QoL was associated with increased lesion load, prolonged working disability and reduced volume in the right thalamus^42,44,45^. However, the results of volumetric analyses of subcortical structures (such as the thalamus in this study) should be interpreted with caution, as usable MRI scans were available for only *n* = 16 subjects (Supplemental Table 1). This limitation did not apply to the volumetric assessment of brain lesions (or “scars in brain tissue”), for which a larger dataset was available (*n* = 82; see Fig. 1). Nevertheless, from acute ischemic stroke-related studies it is evident that particularly right thalamic lesions (with subsequent atrophy) are associated with pain, unlike left thalamic lesions, as the right side is primarily involved in processing pain^45,46^. A tendency toward right thalamic atrophy was also observed in association with reduced psychological quality of life, suggesting a possible relationship between decreased right thalamic volumes and chronic fatigue—a pattern that has been described in the context of post-stroke pain^44,47–50^:

- Overview of Profile S4: “Chronic Pain and Functional Limitations”.
  - Impact of pain on QoL evident, correlating with decreased capacity for daily tasks.
  - Heightened fatigue and psychomotor inhibition due to chronic pain.

Regarding the interpretability of subcortical brain volumes (such as the thalamus), several aspects should be considered, especially in the context of future studies. The data from the present study are not sufficient for definitive conclusions and can only serve as preliminary indications and points for further consideration:

The classification of the negative correlation between the vitality dimension and increased brain parenchyma gray matter volumes in the left pallidum and thalamus should be interpreted with caution due to the limited sample size (*n* = 16). Nonetheless, this association, which reflects psychomotor components, appears to be complex. On the one hand, the enhanced quality of life (QoL) associated with diminished volumes in the thalamus and pallidum could be elucidated by the influence of the profile “stable aneurysms in older adults”: reduced volumes due to physiological aging processes could contribute to an elevated QoL in a demographic typically experiencing minimal concerns related to their aneurysm^51^. On the other hand, psychiatric disorders, such as depression, anxiety disorders, and post-traumatic stress disorder (PTSD), are more common after traumatic experiences^52–54^.However, these disorders typically result in brain parenchymal atrophy of subcortical structures including the thalamus/pallidum rather than volume increase^52–54^. Only isolated studies have described an increase in these structures regarding PTSD^55^. Conversely, after SAH, increased compulsive-like behavioral abnormalities were observed in mice, with significantly increased psychomotor agitation^56^. Similar findings were observed in cases of SAH in humans, where, among other complications, Obsessive-Compulsive Disorder (OCD) developed as a late consequence, leading to disability months after the acute phase^23,57^. OCD is frequently associated with frontal brain damage^58–61^. In approximately 30–50% of reported cases, vasospasms occur after aneurysmal SAH^27,28^, leading to subsequent DCI in the anterior cerebral artery (ACA) territory, resulting in brain lesions over time^22^. Regarding a neuroimaging correlate, OCD appears to be associated with increased volumes in the thalamus and pallidum^62,63^. Thus, this diagnosis as a late consequence after aneurysmal SAH appears to be another risk factor for potential working disability.

Several limitations must be acknowledged. The study included only 111 patients, with lesion volumes available for 82 and brain parenchymal volumes for just 16 subjects, due to the retrospective use of MRI data from various sources. Consequently, analyses involving brain parenchymal volumes could not be generalized to the entire cohort. Because of the small sample size, multiple regression analyses were largely omitted to avoid overfitting; subgroup analyses (e.g., by sex or rupture status) were not feasible, and only a single regression with four predictors was performed for the total population. To identify subgroups, principal component analyses and complementary correlation analyses were used instead. There is a risk of selection bias, as patients without consent to participate—potentially those with more severe disease—were excluded, possibly skewing results toward a healthier population. Future studies should seek ethics approval to include data from severely affected or deceased patients. The predominance of female patients (about 75%) reflects the known epidemiology of cerebral aneurysms but limits generalizability. Additionally, work disability was self-reported via an online questionnaire, raising the possibility of misunderstanding or misreporting, especially among retired or otherwise non-working respondents, which could distort results. Another limitation of this study is that ist was not distinguished whether participants returned to work on a part-time or full-time basis.

## Conclusion

To conclude, this study demonstrated that patients with cerebral aneurysms experience significant reductions in health-related quality of life (QoL), compared to a healthy population. Multimodal analysis revealed that patients with brain aneurysms exhibit a complex relationship between mental and physical quality of life, with mental well-being playing a significant role in disease perception: isolated physical limitations did not contribute significantly to prolonged work disability compared to these factors. Principal component analyses, incorporating clinical, demographic, imaging, and patient-reported variables, enabled the identification of distinct patient profiles that reflect the complex interplay of residual brain damage, age, sex, chronic pain, and psychological stress. Main disease profiles were identified: (1) those with increased LV post-rupture (high DW); (2) older individuals with stable aneurysms (low DW); (3) revealing a sex disparity in QoL despite similar vasospasm severity, whereas women reported lower QoL than men, independent of clinical severity, underscoring the importance of psychosocial factors; and 4), focused on chronic pain and its impact on daily tasks. Two sub-profiles highlighted trauma-induced impairments, functional disabilities from LV, and persistent anxiety. Lastly, a subgroup experiencing a decline in quality of life due to anxiety induced by an untreated aneurysm was identified, although this association was not strongly linked to prolonged work disability. The results of the present study indicate that the quality of life of brain aneurysm patients can be improved by adequately addressing the individual needs of the identified subgroups. A more detailed investigation of the subgroups outlined here needs to be further specified in subsequent publications. Future research should focus on optimizing medical care and enhance physician-patient communication to achieve this objective.

## Supplementary Information

Below is the link to the electronic supplementary material.

Supplementary Material 1

Supplementary Material 2

## Data Availability

Data are available at the data holding institute (Department of Medical Psychology and Medical Sociology of the University Medical Centre Mainz). Inquiries must be sent to the director (K.P.) or the last author of the current study (S.F.). Each request should be based on a scientific hypothesis and have been reviewed by an ethical committee. Any request must be made in writing. Data will be saved for ten years after publication (according to GCP-guidelines), if not declared otherwise by the participants or the legal guardians.

## Abbreviations

1-β: Statistical Power
AI: Artificial Intelligence
aSAH: Aneurysmal Subarachnoid Hemorrhage
B: Regression Coefficient
cMRI: Cerebral Magnetic Resonance Imaging
DCI: Delayed Cerebral Ischemia
DSA: Digital Subtraction Angiography
DW: Disability in Weeks per Year
EmRoFunc: Emotional Role Function
FLAIR: Fluid–Attenuated Inversion Recovery
GenH: General Health
LLMs: Large Language Models
LVV: Large Vessel Vasospasm
MPRAGE: Magnetization Prepared Rapid Acquisition Gradient Echo
MRI: Magnetic Resonance Imaging
Pain: Pain–related Quality of Life
PCA: Principal Component Analysis
PhyFunc: Physical Functioning
PhyRoFunc: Physical Role Function
PsyWB: Psychological Well–Being
PTSD: Post–Traumatic Stress Disorder
R: Regression Coefficient
R^2^: Coefficient of Determination
SAH: Subarachnoid Hemorrhage
SF: 36–Short Form–36 Health Survey
SoFunc: Social Functioning
SPACE: Sampling Perfection with Application optimized Contrasts by Excitation
SPSS: Statistical Package for the Social Sciences
TSE: Turbo Spin Echo
Vit: Vitality
β: Standardized Beta Coefficient
