# Supplementary material for: Profiling disease experience in patients living with brain aneurysms by analyzing multimodal clinical data and quality of life measures
Source: Sci Rep. 2025 Aug 20;15:30562. doi: 10.1038/s41598-025-15544-1 (PMC12368144; doi:10.1038/s41598-025-15544-1)
Supplement: Supplementary file 1 — Supplementary Material 1 [file 41598_2025_15544_MOESM1_ESM.docx]

Supplemental Table 1: Correlation of Physical and Mental Dimensions with Brain Volume.

| Brain Volume [ml] |  | Left Caudate Ncl. | Right Caudate Ncl. | Left Putamen | Right Putamen | Left Pallidum | Right Pallidum | Left Thalamus | Right Thalamus |
| --- | --- | --- | --- | --- | --- | --- | --- | --- | --- |
| Working Inability [Weeks/Year] | R | 0.297 | 0.168 | 0.045 | 0.297 | 0.403 | 0.492 | 0.386 | 0.611 |
|  | P | 0.43 | 0.66 | 0.90 | 0.43 | 0.28 | 0.17 | 0.30 | 0.07 |
| Ruptured Aneurysm (Yes=1) | R | 0.129 | 0.103 | 0.155 | 0.103 | 0.297 | 0.232 | 0.129 | 0.026 |
|  | P | 0.61 | 0.68 | 0.54 | 0.68 | 0.23 | 0.35 | 0.61 | 0.91 |
| Vasospasm Severity (Severe=4) | R | 0.078 | 0.035 | 0.088 | 0.035 | 0.259 | 0.180 | 0.052 | -0.047 |
|  | P | 0.75 | 0.88 | 0.72 | 0.88 | 0.30 | 0.47 | 0.83 | 0.85 |
| Brain Lesion Volume [ml] | R | 0.061 | 0.152 | -0.094 | -0.063 | -0.087 | 0.093 | -0.249 | -0.074 |
|  | P | 0.81 | 0.54 | 0.71 | 0.80 | 0.73 | 0.71 | 0.32 | 0.76 |
| Residual Aneurysm Perfusion | R | -0.131 | -0.060 | -0.167 | -0.131 | -0.299 | -0.299 | 0.131 | 0.108 |
|  | P | 0.60 | 0.81 | 0.50 | 0.60 | 0.22 | 0.22 | 0.60 | 0.67 |
| Physical Domain: Physical Role Function | R | -0.474 | -0.529 | -0.225 | -0.148 | -0.333 | -0.500 | -0.234 | -0.034 |
|  | P | 0.16 | 0.11 | 0.53 | 0.68 | 0.34 | 0.14 | 0.51 | 0.92 |
| Physical Domain: General Health | R | -0.148 | -0.197 | -0.034 | -0.339 | 0.488 | 0.012 | 0.449 | 0.207 |
|  | P | 0.68 | 0.58 | 0.92 | 0.33 | 0.15 | 0.97 | 0.19 | 0.56 |
| Physical Domain: Physical Functioning | R | -0.474 | -0.529 | -0.225 | -0.148 | -0.333 | -0.500 | -0.234 | -0.034 |
|  | P | 0.16 | 0.11 | 0.53 | 0.68 | 0.34 | 0.14 | 0.51 | 0.92 |
| Physical Domain: Pain-related Quality of Life | R | -0.130 | -0.117 | 0.195 | 0.278 | 0.347 | 0.285 | 0.395 | **0.625** |
|  | P | 0.72 | 0.74 | 0.58 | 0.43 | 0.32 | 0.42 | 0.25 | **0.05** |
| Mental Domain: Vitality | R | -0.328 | -0.292 | -0.372 | -0.146 | **-0.741** | -0.463 | **-0.687** | -0.354 |
|  | P | 0.35 | 0.41 | 0.29 | 0.68 | **0.01** | 0.17 | **0.02** | 0.31 |
| Mental Domain: Psychological Well-being | R | -0.122 | -0.158 | -0.040 | 0.249 | 0.299 | 0.265 | 0.322 | **0.591** |
|  | P | 0.73 | 0.66 | 0.91 | 0.48 | 0.40 | 0.45 | 0.36 | **0.05** |
| Mental Domain: Emotional Role Function | R | -0.413 | -0.343 | -0.172 | -0.025 | -0.271 | -0.402 | -0.159 | 0.029 |
|  | P | 0.23 | 0.33 | 0.63 | 0.94 | 0.44 | 0.25 | 0.66 | 0.93 |
| Mental Domain: Social Functioning | R | -0.250 | -0.312 | 0.080 | 0.111 | 0.066 | -0.083 | 0.222 | 0.313 |
|  | P | 0.48 | 0.38 | 0.82 | 0.76 | 0.85 | 0.81 | 0.53 | 0.37 |
