# Supplementary material for: Profiling disease experience in patients living with brain aneurysms by analyzing multimodal clinical data and quality of life measures
Source: Sci Rep. 2025 Aug 20;15:30562. doi: 10.1038/s41598-025-15544-1 (PMC12368144; doi:10.1038/s41598-025-15544-1)

**Supplemental Figure 1:** Regression Analyses predicting the Weeks of Work Disability per years (DW) using age, sex, mental health, and physical health. Unstandardized regression coefficient (B) with 95% confidence interval (CI), standardized beta coefficient (β) with 95% CI, standard error (SE), t-value (T), and significance level (p-value) are reported for each predictor.
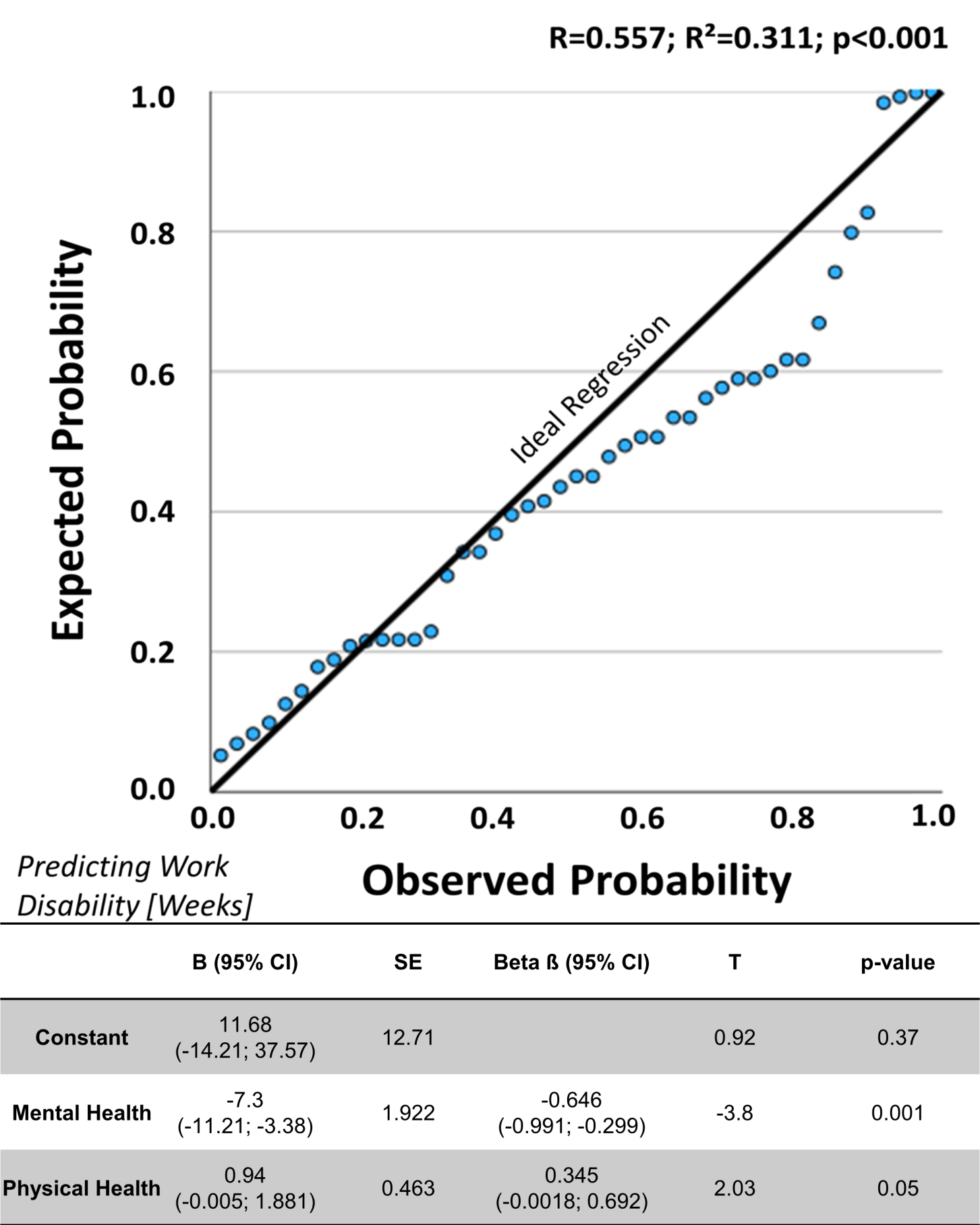

Supplement: Supplementary file 2 — Supplementary Material 2 [file 41598_2025_15544_MOESM2_ESM.docx]
